# News and Miscellany

**Published:** 1905-07

**Authors:** 


### News and Miscellany.

---

### Letter From Dr. Rosser of Dallas.

---

CHICAGO, July 1, 1905.

*Dear Dr. Daniel:*

Since turning my attention more directly to surgical matters some years ago, I have tried annually, or oftener, to look in upon some of the splendid clinics of New York, Philadelphia, Baltimore and Chicago, and to learn what I could of the methods which in the hands of men doing advanced work has made American surgery the surgery of the century. My present vacation having begun in June, I purpose to pass through July and into August, and to devote primarily with Dr. Alexander Hugh Ferguson, at the Chicago hospital, and incidentally in observing other excellent operators here, and then the time fortunately arranged for service with the Mayos at Rochester, Minn., where the father, now aged and inactive, and the sons (W. J. and C. F.), young and vigorous, have established a clinic which on account of location, extent and results, is both remarkable and unique.

I am making notes touching the points which appear either new or otherwise notable and shall continue to jot them down for reference, and if you would like a short service for the JOURNAL I will be glad to give them, if you will indicate the date. Very truly,

CHARLES M. ROSSER.

---

TERRELL, TEXAS, July 4, 1905.

*Dr. F. E. Daniel, Austin, Texas.*

DEAR DOCTOR: In order to testify faithfulness and show my appreciation of *the best medical journal published in the South,* I hand you this $5 exchange for subscription. My regards to the "Mascot." Yours truly,

W. H. MONDAY.

---

"Of all the journals that reach us none is read more regularly or more enjoyably than the TEXAS MEDICAL JOURNAL. You have

*always* wielded a trenchant pen, but as years go by you write better editorials, pithier paragraphs, and, all in all, show that you are keeping to the front in American journalism and setting a pace for others that they no doubt find difficult to keep up with."

SHARP & DOHME.

---

We have journals to burn. The "Red Back" is good enough for me. I prefer the Transactions in one volume. (Renews for 21st year.)

W. J. COLLINS, M. D., The Grove, Texas.

---

SUICIDE OF A DOCTOR IN DALLAS.—Dr. W. S. Leeman, late professor of medicine in the Medical Department of Baylor University, Dallas, committed suicide by shooting July 5th, inst. No cause is known for it. He was 38 years of age, a graduate of St. Louis College of Physicians and Surgeons, and had a good practice.

---

THE HOOK WORM DISEASE (Ankylostomiasis) causes anemia or, rather, is always associated with it. The United States commission to investigate and report on the disease in Porto Rico recommend that, after starving and purging the patient and administering anthelmintics to destroy the parasite, he should have a systematic course of treatment by iron, and recommend Pepto Mangan Gude as the best to produce red corpuscles and rebuild the broken down tissues. The report is exhaustive and intensely interesting, and a copy may be had free from M. J. Breitenbach & Co., 53 Warren St., New York. Read their ad. carefully. The observations of the Commission were made under Government control, and, therefore, the report may be regarded as a supreme test, and the efficacy of Pepto-Mangan in one of the most severe forms of anemia is proved beyond a doubt.

---

ERRATA.—For "The *Reporter*, the journal of the North American Retail Druggists' Association" (lines 8 and 9, page 500, in our June issue), read "N. A. R. D. Notes, the journal of the National Association of Retail Druggists."

---

IT KILLS THEM.—Prof. Allen J. Smith, late Professor of Pathology, Medical Department University of Texas, now of the University of Pennsylvania, put a slide containing a culture of tubercle bacilli in a sealed envelope in the pocket of a coat hanging up in the room; and another he wrapped in two thicknesses of billiard cloth, and turned loose an immense volume of formaldehyde on them. In a very short while it killed them, as it did also the bacilli of diptheria and the sporulating anthrax bacilli, the toughest of all pathological germs. The apparatus used was the Germicide Gas Generator. The formaldehyde is generated rapidly direct from the wood alcohol through a copperized disc. No danger of fire,—a child can operate it,—no keyhole "contrapsion',—no moisture to ruin wall paper. It penetrates mattresses, bedding, carpets, rags, clothing, all *in situ* and thoroughly disinfects everything in a room. Every doctor should own one. Pays for itself by renting it. Every room in which a consumptive lives, or has died, every school room, every church, ward, boarding house, courthouse, jail, and hotel should be disinfected at intervals. Price $25, by express free anywhere in Texas on receipt of price. Literature on request. Address: Dr. F. E. Daniel, Austin, Texas.

---

INSULT TO INJURY.—The Octopus is piling up money, investing it in Chicago rent property and in securities. It has an income of $40,000 a year in excess of expenses. This money belongs to the membership. They are not consulted about its investment. Do they get any dividends? In the issue of July 8th, inst., ye editor gives the readers—whose payments for subscription and the revenue from ads create this excess—pretty pictures of some of its (their) property. What good does the property or the pictures do them? The subscription should be reduced or subscribers should get a rebate, or a dividend. Is the great *Journal A. M. A.* run to buy Chicago property?—*Cui bono?*

---

PERSONAL.—Dr. Jno. O. McReynolds and family, Dallas, have returned from Europe.

---

IN THE CASE OF W. W. Stone, Jackson County, who was fined $50 for practicing without a license and who appealed from the decision, the Court of Criminal Appeals held that the law is constitutional.

---

THE NEXT MEETING of the State Board of Medical Examiners of Texas (regular) will be held at San Antonio October 7, 8, 9. Dr. T. T. Jackson, San Antonio, is Secretary, and Dr. S. R. Burroughs, of Buffalo, is President.

Dr. F. B. Maner, of Itasca, Hill ounty, was killed June 26th inst., in a street duel with E. E. Griffin.

